# The impact of technostress on female teachers’ perceived career progression: the mediating role of work–family conflict

**DOI:** 10.3389/fpsyg.2026.1881723

**Published:** 2026-08-20

**Authors:** Li Fu

**Affiliations:** School of Marxism, Zhengzhou Tourism College, Zhengzhou, Henan, China

**Keywords:** higher education institutions, job demands-resources theory, perceived career progression, technostress, work–family conflict

## Abstract

**Introduction:**

This study examines the relationship between technostress, work–family conflict (WFC), and female teachers’ perceived career progression in Chinese higher education institutions in Henan Province, China. As universities increasingly rely on digital technologies for teaching, administration, and communication, female teachers may experience growing technological demands alongside existing professional and family responsibilities. Drawing on the Job Demands-Resources (JD-R) model and Conservation of Resources (COR) theory, this study investigates whether WFC mediates the relationship between technostress and perceived career progression.

**Methods:**

Data were collected from 466 female teachers employed in higher education institutions in Henan Province, China, using a well-structured questionnaire. The data were then analyzed using SPSS and SmartPLS to test the proposed hypotheses.

**Results:**

The results indicate that technostress has a negative relationship with female teachers’ perceived career progression, while it has a positive relationship with work–family conflict. In addition, work–family conflict acts as a mediator in the relationship between technostress and perceived career progression.

**Discussion:**

These findings suggest that technostress has a negative influence on teachers’ perceived career progression in higher education institutions by intensifying work–family conflict. This is consistent with the United Nations Sustainable Development Goals (SDGs) 3, 4, and 5, highlighting the importance of promoting teacher well-being, enhancing educational quality, and advancing gender equality. The paper also offers practical implications for policymakers to alleviate technostress and work–family conflict, thereby supporting teachers’ sustainable perceived career progression.

## 1 Introduction

Limited perceived career progression among female teachers has become an increasingly important concern in higher education institutions worldwide. Although women’s participation in academia has increased substantially over recent decades, they continue to be underrepresented in senior academic and leadership positions across many national contexts (Fernandez et al., 2024; Araneda-Guirriman et al., 2023). Existing studies suggest that female faculty members often encounter structural and institutional barriers related to promotion opportunities, professional recognition, workload distribution, and work-life balance, all of which may restrict career development (Mayya et al., 2021; Wang and Gao, 2024). These concerns have become increasingly important because universities now rely heavily on female teachers to support teaching quality, research productivity, and institutional development.

**Figure 1:**
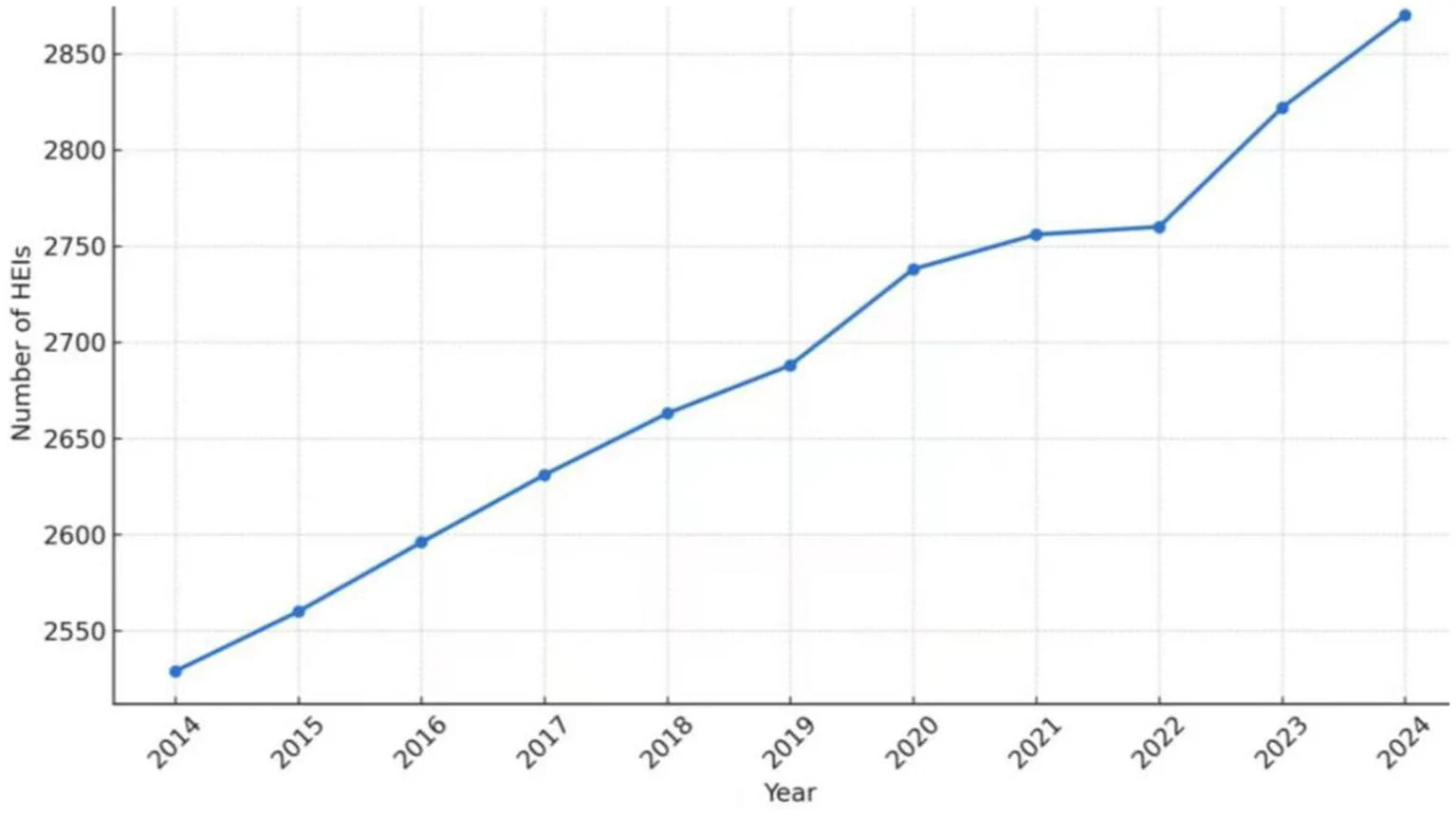
Number of HEIs in China, 2012–2024 (Ministry of Education of the People’s Republic of China, 2024).

In China, the expansion of higher education has accelerated considerably during the past decade. As shown in Figure 1, the number of higher education institutions has steadily increased between 2012 and 2024, reflecting the rapid growth of the higher education sector and the increasing demand for academic labor. At the same time, universities have undergone substantial digital transformation, with online teaching systems, digital administrative platforms, and technology-based performance evaluation becoming increasingly integrated into academic work. While these changes may improve educational efficiency and institutional management, they also create additional technological demands for university teachers.

**Figure 2:**
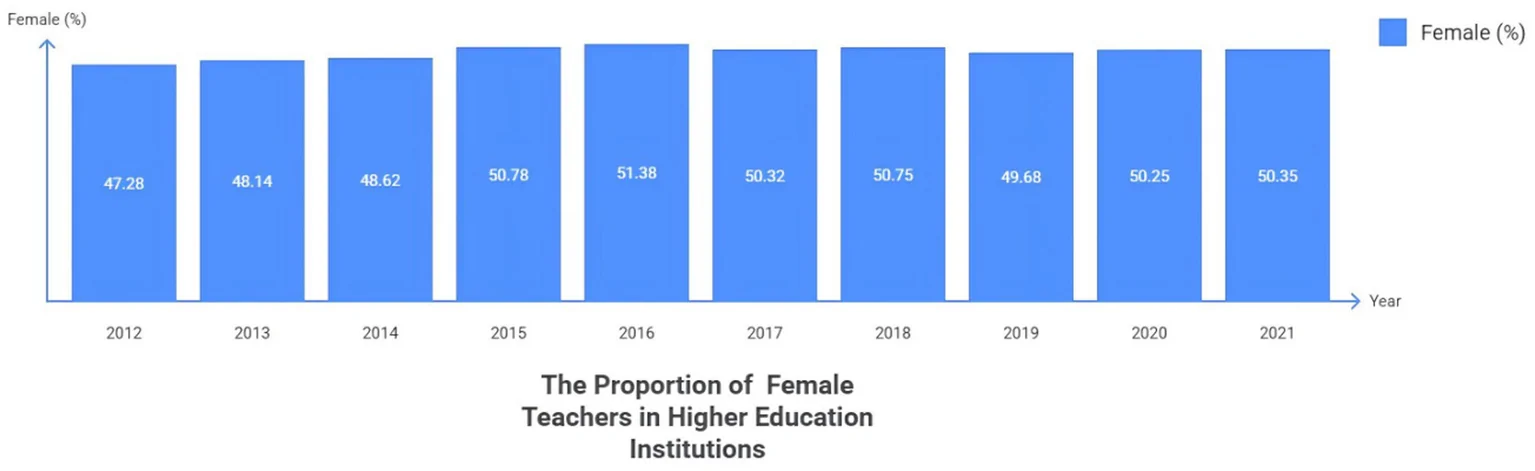
The proportion of female teachers in HEIs in China, 2012–2021 (Ministry of Education of the People’s Republic of China, 2022).

The participation of female teachers in Chinese higher education has also remained consistently high. As illustrated in Figure 2, female faculty members have accounted for nearly half of academic staff in Chinese higher education institutions over the past decade. Despite this increasing participation, women remain underrepresented in senior academic positions and leadership roles. Existing studies suggest that numerical participation does not necessarily translate into equal career advancement opportunities, particularly within highly competitive academic environments characterized by increasing research expectations and performance pressures (Khosa, 2023).

**Figure 3:**
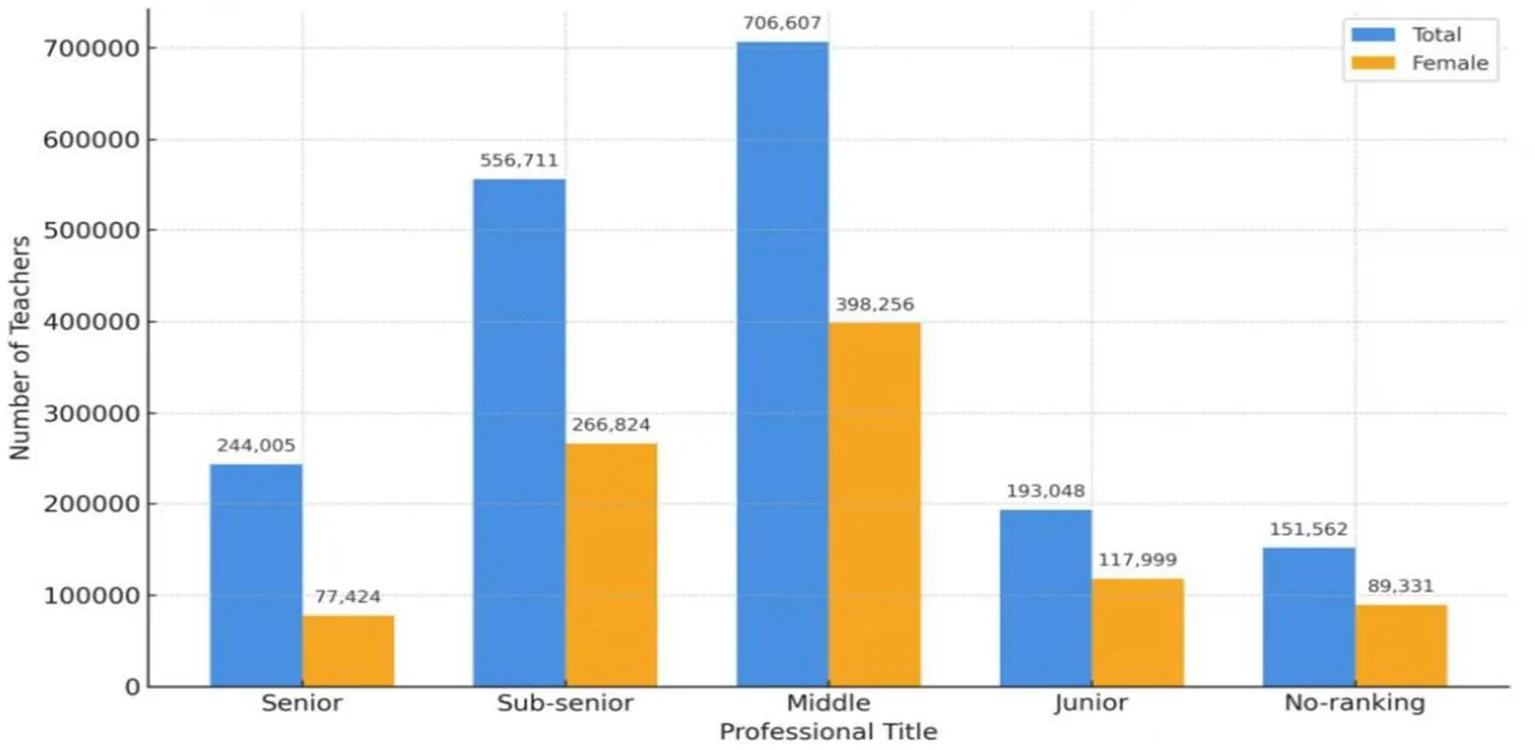
Academic ranks of female teachers in HEIs in China (Ministry of Education of People’s Republic of China, 2024).

At the same time, female teachers continue to face challenges related to perceived career progression within academic ranking systems. As shown in Figure 3, female teachers remain concentrated in junior and intermediate academic ranks, while representation at senior levels remains comparatively limited. This pattern suggests that female teachers may encounter barriers that extend beyond participation itself and involve broader institutional, professional, and social pressures affecting perceived career progression.

One increasingly important challenge in this context is technostress. The rapid digitalization of higher education has substantially transformed academic work, requiring teachers to adapt continuously to online teaching platforms, digital administrative systems, virtual communication technologies, and technology-based performance management. Although these technologies may improve flexibility and efficiency, they may also generate excessive technological demands, continuous connectivity, cognitive overload, and blurred work-life boundaries (Khlaif et al., 2023; Pansini et al., 2023). In academic settings, teachers are frequently expected to remain digitally responsive beyond regular working hours while simultaneously maintaining teaching quality, research productivity, and administrative responsibilities. Under such conditions, technology-related demands may gradually consume individuals’ cognitive and emotional resources and negatively affect their professional development.

These pressures may be particularly significant for female teachers, who often manage both professional responsibilities and family caregiving roles simultaneously. In many social and cultural contexts, women continue to assume primary household and caregiving responsibilities, making them more vulnerable to work–family conflict (WFC) when job demands intensify (Mahapatra and Ford, 2024). The integration of digital technologies into academic work has further blurred the boundaries between work and family domains by extending work-related responsibilities into personal time. As a result, female teachers may experience greater difficulty balancing teaching, research, administrative tasks, and family obligations. Persistent exposure to these competing demands may reduce the time, energy, and psychological resources available for career-related activities such as academic networking, publication preparation, leadership participation, and professional development.

Drawing on the job demands-resources (JD-R) model, technostress can be understood as a job demand that requires sustained cognitive and emotional effort and may lead to strain and resource depletion when institutional support is insufficient (Bakker and Demerouti, 2017). Conservation of Resources (COR) theory further suggests that individuals attempt to preserve limited personal resources such as time, energy, and emotional stability, and stress occurs when these resources are threatened or depleted (Hobfoll, 1989). Within this perspective, continuous technological demands may intensify WFC by extending work responsibilities into family life and increasing role-related pressures. Over time, the depletion of personal resources may negatively influence female teachers’ motivation, wellbeing, and capacity to pursue perceived career progression.

Technostress has attracted considerable scholarly attention for its associations with burnout, job satisfaction, and psychological wellbeing (Herrera-Sánchez et al., 2024; Borle et al., 2021; Ramesh et al., 2022), yet existing studies concentrate mainly on immediate outcomes such as emotional exhaustion and job dissatisfaction (Yener et al., 2020; Sharma and Tiwari, 2023), leaving its implications for perceived career progression largely unexplored. This is a notable omission: persistent technology-related demands may gradually deplete the time, energy, and cognitive resources that underpin professional advancement. Prior studies have reported a relationship between technostress and work–family conflict (Finstad et al., 2024; Camacho and Barrios, 2022). However, less is known about whether work–family conflict explains the association between technostress and career-related outcomes. This issue is theoretically relevant because, according to Conservation of Resources theory, resources depleted by workplace stressors may affect not only employees’ immediate well-being but also their capacity to engage in career-related activities. Furthermore, teachers are typically studied as a homogeneous occupational group, overlooking gender-specific barriers documented among Chinese female academics (Wei et al., 2025), and empirical evidence from inland China remains scarce despite rapid educational digitalization, with Dong et al. (2025) among the few studies addressing Henan Province specifically. This study addresses these gaps by examining the association between technostress and female teachers’ perceived career progression through the mediating role of work–family conflict in Henan Province, extending technostress research beyond short-term psychological outcomes through a gender-sensitive, resource-based perspective grounded in an underrepresented regional context.

These issues may be particularly relevant in Henan Province. Regional disparities in Chinese higher education are reflected not only in funding differences but also in measurable indicators of institutional development. Using QS World University Rankings data to construct province-level density and quality indices, Wu and Borhan (2024) reported that Henan, despite being one of China’s most populous provinces, had no institution included in the QS World University Rankings until 2022, and its composite higher education index relative to Beijing was only 0.0108 in 2022–2023. Previous studies have also shown that inequalities between coastal and inland regions are evident in technology application, resource allocation, and digital infrastructure development (Huang, 2024; Xie and Zhang, 2024). These institutional differences may influence the everyday work experiences of female academics. Compared with universities in more developed coastal regions, institutions with fewer research resources and more limited investment in digital infrastructure may face greater challenges in providing technical support, digital training opportunities, and workload adjustments during technological transitions. Consequently, digital transformation may create additional technology-related demands for female teachers working in inland higher education contexts, where institutional resources for managing such demands may be relatively constrained.

This study advances the existing literature in three ways. First, while previous technostress research has primarily focused on immediate psychological and occupational outcomes, this study extends current understanding by examining its association with a broader career-related outcome, namely perceived career progression. This perspective complements the JD-R model by considering how technology-related demands may influence outcomes beyond immediate strain. Second, this study places female teachers at the center of analysis, addressing the limited attention given to gender-specific experiences in technostress research and recognizing the distinct career challenges faced by women in academia. Third, by examining higher education institutions in Henan Province, China, this study expands the geographical scope of technostress research beyond the contexts that have received greater empirical attention and provides evidence from an underrepresented regional setting.

Against this background, the present study examines the relationship between technostress, work–family conflict, and female teachers’ perceived career progression in Chinese higher education institutions, drawing on the JD-R model and Conservation of Resources theory to investigate whether work–family conflict mediates this relationship among female teachers in Henan Province, China. Accordingly, this study examines the relationships among technostress, work–family conflict, and perceived career progression among female teachers in higher education institutions in Henan Province, China. Drawing on the Job Demands-Resources model and Conservation of Resources theory, the study addresses the following research questions:

*RQ1*: How is technostress associated with female teachers’ perceived career progression?

*RQ2*: How is technostress associated with work–family conflict among female teachers?

*RQ3*: Does work–family conflict mediate the association between technostress and female teachers’ perceived career progression?

Addressing these questions also contributes to the United Nations Sustainable Development Goals (SDGs), particularly SDG 3 (Good Health and Well-being), SDG 4 (Quality Education), and SDG 5 (Gender Equality). By identifying how technology-related work demands are associated with female teachers’ work-family experiences and perceived career progression, this study offers evidence relevant to more equitable and sustainable higher education workplaces.

## 2 Literature review and hypotheses development

### 2.1 Technostress

Technostress refers to the stress individuals experience when coping with technology-related demands in the workplace. In higher education, the rapid expansion of digital teaching platforms, online administrative systems, and virtual communication has increased teachers’ reliance on information and communication technologies. Although these technologies improve teaching efficiency and institutional management, they also increase workload and blur the boundaries between work and personal life (Siddiqui et al., 2023; Khlaif et al., 2023). Previous studies have shown that prolonged technological demands may contribute to emotional exhaustion, reduced well-being, and higher levels of work–family conflict among university teachers (Estrada-Muñoz et al., 2020; Kulikowski et al., 2022). These challenges may be particularly significant for female teachers, who often face additional family responsibilities alongside academic work.

Empirical work situates technostress within the Job Demands-Resources framework as a job demand linked to psychological distress, emotional exhaustion, and reduced wellbeing, with these effects intensifying when organizational support is low (Pansini et al., 2023; Harunavamwe and Ward, 2022; Panisoara et al., 2020; Ghislieri et al., 2022). Studies conducted during COVID-19 similarly link technostress to elevated work–family conflict and diminished wellbeing, with women’s greater caregiving burdens intensifying these effects and accentuating perceived career progression risks absent sponsor support and family-friendly policies (Shaukat et al., 2022; Wang et al., 2023; Mahapatra and Ford, 2024). Beyond psychological strain, technostress has also been linked to declining job performance and research productivity over time, with work–family conflict repeatedly identified as the mechanism through which it constrains opportunity-rich activities such as networking and promotion-relevant output (Pansini et al., 2023; Qin et al., 2025; Wang et al., 2023; Harunavamwe and Ward, 2022).

### 2.2 Work–family conflict

Work–family conflict (WFC) refers to a form of inter-role conflict in which the demands of work and family roles are mutually incompatible, making participation in one domain more difficult due to involvement in the other (Greenhaus and Beutell, 1985). It encompasses bidirectional processes, namely work-to-family conflict and family-to-work conflict, and manifests in three distinct forms: time-based, strain-based, and behavior-based conflict.

In China, traditional gender norms and institutional pressures have amplified WFC among female teachers, as Confucian cultural values primarily assign caregiving responsibilities to women, exacerbating resource depletion when combined with demanding academic roles (Chakravorty and Singh, 2021; Escudero-Guirado et al., 2024). Recent policy shifts, such as the three-child policy, have been associated with increased caregiving expectations, further limiting the time and energy available for academic work and heightening the risk of WFC among women in academia (Simães et al., 2021; Ma et al., 2025). Multiple job demands including teaching, research, and administration, compounded by ICT-driven responsibilities, exacerbate boundary permeability between work and family domains, accelerate resource depletion, and disproportionately affect the careers of women who lack mentorship or family-friendly institutional support (Cao and Zhang, 2022; Ghislieri et al., 2025; Kim and Yeo, 2024).

### 2.3 Female teachers’ perceived career progression

Perceived career progression, broadly defined by Super (1980), refers to the lifelong developmental process through which individuals advance in occupational roles, responsibilities, and status. Baruch et al. (2025) later redefined perceived career progression as a boundaryless, multi-directional structure that includes objective outcomes (such as promotion, salary) and subjective evaluations (such as job satisfaction, perceived growth), which still holds influence in contemporary career research. In the context of education, the perceived career progression of female teachers is particularly prominent, as they encounter disproportionate gender structures and cultural barriers in the promotion process (Dehghanpour-Farashah et al., 2025). According to Koontz et al. (2019), female teachers’ perceived career progression is shaped by the interplay of organizational support, professional identity, and family demands, with work–family conflict frequently cited as a significant impediment. However, there is no unified definition of female teacher perceived career progression in the literature, as its determinants and expressions may vary across educational systems, cultural backgrounds, and career stages. Therefore, this study adopts the Job Demands-Resources (JD-R) (Bakker and Demerouti, 2017) to view the perceived career progression of female teachers as the result of a dynamic balance between job demands and available resources, where sustained perceived career progression is influenced by an individual’s ability to manage technostress and work–family conflicts under sustained work pressure conditions (Du et al., 2025).

Previous studies on female teacher perceived career progression have primarily focused on organizational, institutional, and individual factors such as mentorship, leadership style, and self-efficacy, with limited attention to technology-induced stressors or the mediating role of work–family conflict in shaping career outcomes (Kamalu and Kamalu, 2021; Choge, 2023). Moreover, most existing research has treated teachers as a homogeneous group, overlooking gender-specific challenges that female teachers face in balancing technological demands with family responsibilities (Ryan et al., 2021). In China, research on technostress among educators has largely focused on its effects on job satisfaction and burnout, while its impact on perceived career progression, particularly for female teachers, remains underexplored.

### 2.4 An integrated JD-R-COR mechanism linking technostress to perceived career progression

The relationships among technostress, work–family conflict, and perceived career progression can be better understood through an integrated perspective of the Job Demands-Resources (JD-R) model and Conservation of Resources (COR) theory. From the JD-R perspective, technostress represents a significant job demand that requires continuous cognitive and emotional effort to manage (Bakker and Demerouti, 2017). However, while the JD-R model explains why excessive job demands may lead to strain, it provides less insight into how depleted resources influence outcomes across different life domains. COR theory complements this perspective by explaining the process of resource loss. Hobfoll (1989) argues that individuals strive to preserve valuable resources, including time, energy, attention, and emotional capacity. When these resources are continuously consumed by workplace demands, individuals may experience difficulty maintaining performance across other important roles.

In the context of female teachers, the influence of technostress can be understood as a resource-depletion process involving sequential effects. First, technology-related demands, including techno-overload, techno-invasion, and techno-complexity, require teachers to invest additional cognitive effort and time in managing digital platforms, administrative systems, and continuous online connectivity (Pansini et al., 2023). According to COR theory, these additional demands may reduce the resources available for fulfilling family responsibilities, thereby increasing work–family conflict. This effect may be particularly relevant for female academics because women often continue to assume greater family and caregiving responsibilities (Mahapatra and Ford, 2024). As work and family boundaries become increasingly blurred, technology-related demands may intensify conflicts between professional and family roles (Shaukat et al., 2022).

Second, work–family conflict may represent an important pathway through which technostress influences perceived career progression. Career advancement requires sustained investment in activities such as research productivity, professional networking, mentoring, and leadership participation, all of which depend on sufficient time and psychological resources (Wang et al., 2023). When resources are consumed by managing technological demands and resolving work-family tensions, female teachers may have fewer resources available for career-related activities. Therefore, technostress may influence perceived career progression not only through direct strain but also through the indirect effects of resource depletion and work–family conflict.

This integrated JD-R and COR perspective provides two important theoretical implications. First, work–family conflict is not merely an outcome associated with technostress but a potential mechanism explaining how technology-related demands translate into career-related consequences. Second, considering the additional family responsibilities often experienced by female academics, the resource-depletion process may be particularly relevant for understanding their career experiences. Based on this theoretical reasoning, the following hypotheses are proposed.

*H1*: Technostress has a negative relationship with female teachers’ perceived career progression.

*H2*: Work–family conflict has a negative relationship with female teachers’ perceived career progression.

*H3*: Work–family conflict mediates the relationship between technostress and female teachers’ perceived career progression.

## 3 Methods

### 3.1 Participants

The target of 500 questionnaires was established before data collection on the basis of statistical and fieldwork considerations. First, an *a priori* power analysis was conducted using G*Power 3.1 (Faul et al., 2009) for a linear multiple regression model with two predictors, corresponding to the endogenous construct with the largest number of direct predictors in the proposed model. Assuming a medium effect size ( $f^{2}=0.15$ ), an alpha level of 0.05, and a statistical power of 0.95, the minimum required sample size was 107. The assumed effect size was selected because the study examined relationships among established organizational and psychological constructs for which small-to-moderate effects are commonly expected.

Second, the planned sample was assessed with reference to PLS-SEM guidance, which recommends considering both model complexity and the need for stable parameter estimates (Hair et al., 2019). Although the minimum sample requirement was substantially lower, a target of 500 was adopted to allow for incomplete questionnaires and to obtain responses from female teachers working in different types of higher education institutions across Henan Province. Based on the anticipated rate of unusable or incomplete questionnaires, this target was expected to yield an adequate final analytical sample. A total of 466 valid questionnaires were retained after data screening, exceeding the minimum sample size indicated by the power analysis and supporting the subsequent PLS-SEM analysis.

There are three main reasons for choosing Henan. Firstly, as one of the provinces with the largest population and over 170 higher education institutions, it has experienced rapid expansion while undergoing national education digitization, which has put enormous technological demands on teachers, making it an appropriate background for researching technological pressure and the perceived career progression of female teachers (Sun et al., 2023). Secondly, previous research has mainly focused on developed coastal areas such as Beijing, Shanghai, and Guangdong, while research in central provinces such as Henan is not yet in-depth enough, which makes this study able to fill the gap in geographical research. Thirdly, practical considerations such as obtaining support from participants and institutions to ensure efficient and reliable data collection further demonstrate that Henan is a suitable research location.

A convenience sampling method was adopted to recruit participants, given the practical challenges of obtaining a complete sampling frame of female teachers across higher education institutions in Henan Province. Institutions were purposively selected to ensure diversity in institution type (public vs. private) and geographic region within the province, and within each institution, questionnaires were distributed to all female teachers who were willing to participate. Although this non-probability sampling approach may limit generalizability (addressed in the Limitations section), it was deemed appropriate given the exploratory and prediction-oriented nature of this study (Hair et al., 2019).

Data were collected between November 2024 and February 2025 from higher education institutions in Henan Province through Wenjuanxing and questionnaires distributed in person; confidentiality was guaranteed, and participation was voluntary and limited to female teachers in higher education institutions. Written informed consent was obtained from all participants prior to data collection. As the original scales were developed in English, they were translated into Chinese and back-translated into English by two bilingual researchers independently to ensure semantic equivalence, following established procedures (Brislin, 1970). Out of 500 distributed questionnaires, 478 were returned (response rate of 95.6%), of which 12 were excluded due to substantial missing data or identical responses across all items, resulting in 466 valid questionnaires (93.2%) retained for analysis.

### 3.2 Measures

The following scales have been adjusted according to research needs and are all 5-point Likert type scales, using anchors 1 (strongly disagree) to 5 (strongly agree).

#### 3.2.1 Technostress

We measured technostress using ten items from the Technostress Creator Scale (TCS) (Ragu-Nathan et al., 2008) (Appendix 1). This scale can be utilized to assess different dimensions of technostress, including techno-overload, techno-invasion, techno-complexity, techno-insecurity, and techno-uncertainty, which represent key sources of technology-related strain in the workplace. Example items include: “I am forced by this technology to work much faster”; and “I am forced by this technology to do more work than I can handle” (Cronbach’s Alpha = 0.82).

#### 3.2.2 WFC

Work–family conflict was measured using an adapted 11-item scale based on Carlson et al. (2000) (Appendix 2). The scale covered work-to-family conflict and family-to-work conflict across time-based, strain-based, and behavior-based conflict. Most items referred broadly to family activities, family responsibilities, or family life. Example items include “My work prevents me from spending sufficient quality time with my family” and “It is difficult to concentrate at work because I am so exhausted by family responsibilities.” The Cronbach’s alpha for the scale was 0.90.

For the purpose of interpreting the broadly worded items, “family” was understood to include nonwork responsibilities and relationships involving parents and other close family members, as well as spouses or partners and children where applicable. However, one item explicitly referred to becoming a better “parent or spouse,” and this item may have been less applicable to respondents who were unmarried or did not have children. Marital status was reported descriptively but was not included as a control variable or examined through a sensitivity analysis. This issue is therefore acknowledged as a limitation of the study.

#### 3.2.3 Perceived career progression

Perceived career progression was assessed using the Career Success Scale (CSS) developed by Judge et al. (1995) (Appendix 3). The scale captures respondents’ subjective evaluations of their career achievements and current career standing, rather than objective indicators of career advancement, such as promotion, academic rank, salary, or changes in responsibility over time. In the present study, perceived career progression therefore refers to female teachers’ self-reported assessment of their career-related achievement and progress at the time of the survey. This operationalization was appropriate for the cross-sectional design because the study examined associations between technostress, work–family conflict, and respondents’ contemporaneous evaluations of their career progress. It does not, however, provide evidence of actual career mobility or longitudinal change. Subjective and objective career success are related but distinct dimensions of career outcomes and may be shaped by different employment conditions (Abele and Spurk, 2009). Example items include “I am satisfied with the success I have achieved in my career” and “I am satisfied with the perceived career progression I have made toward meeting my goals for the development of new skills.” Cronbach’s alpha for the scale was 0.90.

### 3.3 Data analysis

Descriptive statistics were analyzed using SPSS 26.0. The hypothesized relationships were tested using partial least squares structural equation modeling (PLS-SEM) with SmartPLS 4.0. PLS-SEM was considered appropriate because the study focused on prediction-oriented analysis and mediation relationships among latent constructs (Hair et al., 2019). The measurement model was first evaluated in terms of reliability and validity prior to structural model assessment. Bootstrapping with 5,000 resamples was conducted to test the significance of direct and indirect effects. All constructs were modeled as reflective first-order constructs (Sarstedt et al., 2019).

## 4 Results

### 4.1 Demographic profile

As shown in Table 1, most respondents were aged between 36 and 45 years, and the majority were married (84%). Most participants had one or two children and held a Master’s degree (49%). Lecturers represented the largest academic rank group (52%), while most respondents had 4–10 years of teaching experience (34%). In addition, the largest proportion worked in public universities (56%), and half reported a monthly income between RMB 5,000 and RMB 8,000. Overall, the sample reflected diverse demographic characteristics.

**Table 1:** Respondents’ demographic profile.

Demographic profile of respondents (*N* = 466)			
Demographic factors	Category	Frequency (*n*)	Percentage (%)
Age	Below 30	20	5
	31–35	79	17
	36–40	117	25
	41–45	113	24
	46–50	84	18
	Above 50	53	11
Marital status	Single	41	9
	Married	391	84
	Divorced	25	5
	Widowed	9	2
No. of children	0	60	13
	1	180	39
	2	211	45
	More than 2	15	3
Highest educational level	Bachelor’s degree	72	16
	Master’s degree	230	49
	Doctoral degree	164	35
Academic rank	Associate lecturer	58	12
	Lecturer	242	52
	Associate professor	126	27
	Professor	40	9
Years of teaching experience	1–3 years	46	10
	4–10 years	159	34
	11–20 years	140	30
	Above 20 years	121	26
Type of institution	Public university	261	56
	Private university	205	44
Monthly income (RMB)	Under 5,000 RMB	64	14
	RMB 5000–RMB 8000	234	50
	RMB 8001–RMB 11000	115	25
	above RMB 11000	53	11
Total		466	100

Age, academic rank, and teaching experience are relevant contextual characteristics because they may be associated with teachers’ exposure to digital work demands, family responsibilities, and perceptions of career-related achievement. For example, academic rank and teaching experience may reflect differences in work roles, access to institutional resources, and career-stage expectations. In the present study, however, these variables were collected to describe the composition of the sample and were not included as control variables in the structural model. The analysis was designed to test the hypothesized associations among technostress, work–family conflict, and perceived career progression. Their potential role as antecedents, controls, or moderators should be examined in future research.

**Figure 4:**
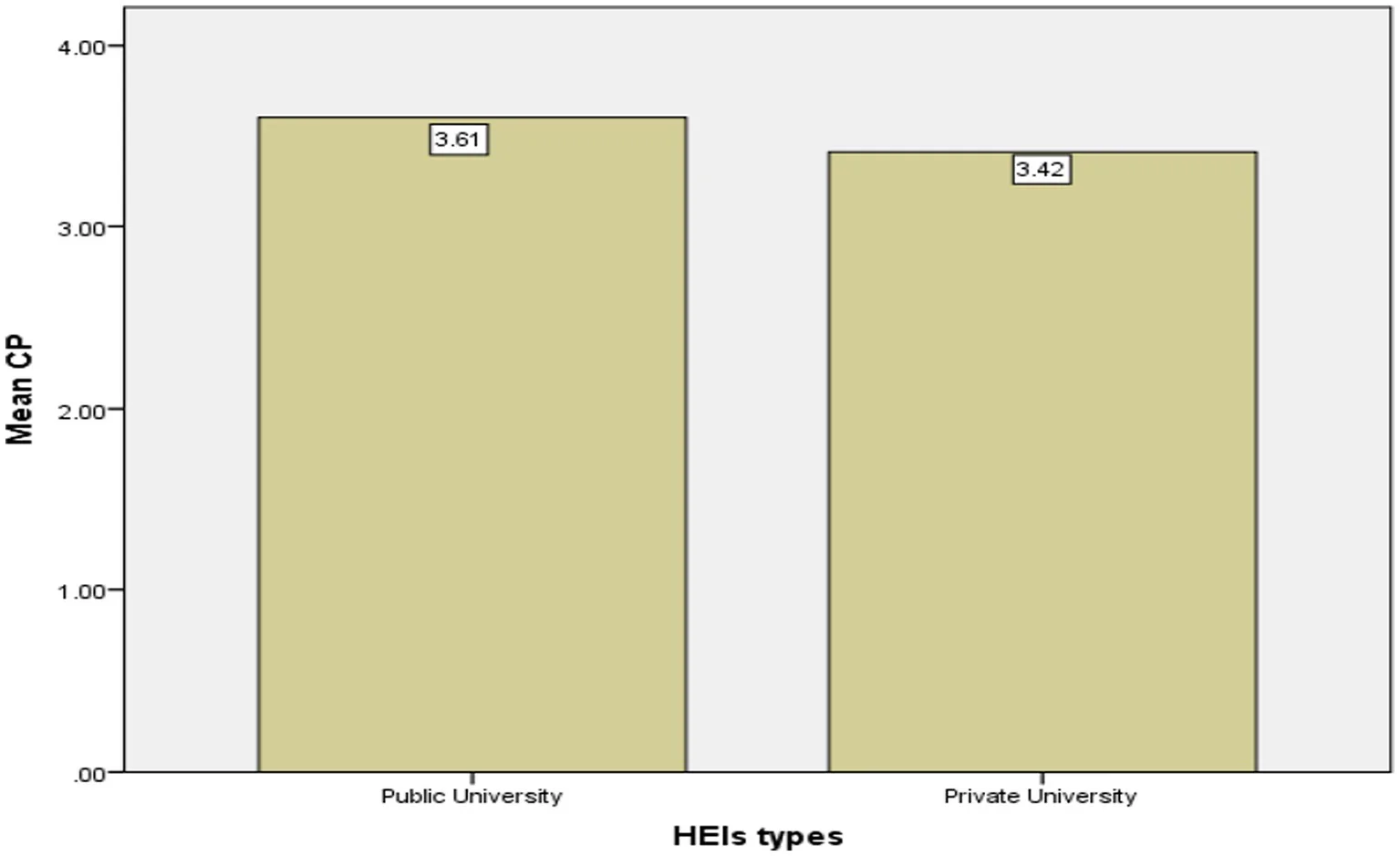
Mean perceived career progression scores across HEI types.

Figure 4 shows variation in female teachers’ perceived career progression across institution types. Female teachers employed in public universities reported higher mean perceived career progression scores (M = 3.61) than those employed in private universities (M = 3.42). This difference may reflect variations in institutional resources, promotion systems, and long-term career support across institution types in Chinese higher education.

These findings are consistent with the research findings of Qin et al. (2019), who found that female teachers in public education institutions typically have access to more structured perceived career progression pathways, including clearer promotion criteria, government funded training programs, and institutional mentorship systems, all of which contribute to upward career mobility. In contrast, private higher education institutions lack such systematic support, which poses significant obstacles to perceived career progression. Mushtaque et al. (2022) further support this view by demonstrating that technostress is particularly evident among teachers in private institutions, where rapid adoption of technology is often mandated without sufficient training or technical support, exacerbating cognitive overload and reducing time available for career advancement activities. The increased technological pressure experienced by female teachers in private higher education institutions not only reduces their professional engagement, but also exacerbates conflicts between work and family, as the ongoing pressure to adapt to new technologies extends working hours to personal time (Shaukat et al., 2022). In addition, Saleem and Malik (2023) point out that technological pressure significantly reduces job satisfaction and organizational commitment, both of which are key drivers of perceived career progression. When female teachers feel a decrease in satisfaction and institutional attachment due to the pressure caused by technology, their willingness and ability to pursue promotion and leadership roles will significantly decrease. In addition, Tu et al. (2025) found that work–family conflict has a disproportionate impact on the perceived career progression of female teachers, as women who struggle to balance technical work demands and family responsibilities are more likely to give up perceived career progression opportunities, exacerbating the negative cycle between technical pressure, work–family conflict, and career stagnation.

Private higher education institutions in Henan Province generally face problems such as unstable employment, heavy workload, and limited institutional resources, as they mainly rely on tuition income rather than government subsidies. Compared to teachers in public universities, teachers in private higher education institutions, especially female teachers, face enormous performance evaluation pressure and need to adapt to rapidly developing digital teaching platforms. However, at the same time, their salary levels are low and perceived career progression opportunities are limited. Due to the lack of systematic technical training in private higher education institutions, female teachers must independently operate complex digital tools, which greatly exacerbates their technical pressure and has a negative impact on work family balance and perceived career progression. In contrast, female teachers in public universities in Henan Province enjoy higher employment security, stable salaries, government supported professional development projects, and more complete technical infrastructure and training support, all of which help alleviate technological pressure and reduce conflicts between work and family. In addition, the clearer career promotion standards and more comprehensive institutional mentorship system in public universities provide female teachers with career direction and a sense of achievement, which helps them achieve sustainable perceived career progression. Compared to female teachers in private universities in Henan Province, these factors have resulted in less negative impact of technostress on the perceived career progression of female teachers in public universities.

### 4.2 Common method variance

Given that all data were collected from a single source at one point in time, several measures were taken to detect and mitigate common method bias (CMB). As an initial diagnostic step, an exploratory factor analysis using Harman’s single-factor extraction was performed on all items measuring technostress, work–family conflict, and perceived career progression. The unrotated solution revealed that the first factor accounted for 42.10% of the total variance (Table 2), which falls well below the critical 50% threshold, suggesting that CMB does not pose a serious threat to the data (Podsakoff et al., 2003). To further assess the possibility of common method bias, variance inflation factor (VIF) values were examined for all measurement items. The VIF values ranged from 1.590 to 2.301 (Table 3), remaining below the recommended threshold of 3.3, suggesting that multicollinearity and common method bias were unlikely to affect the results (Kock, 2015). Beyond these statistical checks, several procedural safeguards were incorporated into the survey design: respondents were assured of complete anonymity, the questionnaire clearly stated that no responses would be judged as correct or incorrect, and items belonging to different constructs were presented in a counterbalanced order to reduce consistency bias (Chang et al., 2019).

**Table 2:** Harman’s single-factor test.

Component	Initial eigenvalues			Extraction sums of squared loadings		
	Total	% of variance	Cumulative %	Total	% of variance	Cumulative %
1	10.945	42.096	42.096	10.945	42.096	42.096
2	3.031	11.658	53.754	3.031	11.658	53.754
3	1.263	4.857	58.611	1.263	4.857	58.611
4	0.688	2.647	61.258			
5	0.656	2.252	63.799			

**Table 3:** Measurement model result.

Construct/items	Loadings	*α*	CR	AVE	VIF
Technostress		0.922	0.935	0.589	
TS-1	0.788				2.192
TS-2	0.753				1.983
TS-3	0.764				1.998
TS-4	0.788				2.157
TS-5	0.776				2.072
TS-6	0.757				1.973
TS-7	0.771				2.028
TS-8	0.75				1.913
TS-9	0.752				1.927
TS-10	0.775				2.052
Work–family conflict		0.926	0.937	0.575	
WFC-1	0.754				1.978
WFC-2	0.754				1.973
WFC-3	0.733				1.824
WFC-4	0.793				2.227
WFC-5	0.747				1.943
WFC-6	0.799				2.301
WFC-7	0.744				1.960
WFC-8	0.772				2.107
WFC-9	0.742				1.870
WFC-10	0.74				1.929
WFC-11	0.76				2.047
Female teacher perceived career progression		0.824	0.876	0.587	
FTPPCP-1	0.763				1.590
FTPPCP-2	0.77				1.675
FTPPCP-3	0.778				1.651
FTPPCP-4	0.755				1.575
FTPPCP-5	0.764				1.621

### 4.3 Measurement model

The measurement model was assessed in terms of indicator reliability, internal consistency reliability, convergent validity, and discriminant validity. As shown in Table 3, all outer loadings exceeded the recommended threshold of 0.70, ranging from 0.733 to 0.799. Cronbach’s alpha values ranged from 0.824 to 0.926, while composite reliability (CR) values ranged from 0.876 to 0.937, indicating satisfactory internal consistency reliability (Table 4).

**Table 4:** Evaluation criteria for the measurement model.

Category	Threshold	Sources
Outer loading	≥0.7	Chin (1998)
Cronbach’s alpha (*α*)	≥0.7	Cronbach (1951)
Composite reliability (CR)	≥0.7	Hair et al. (2019)
Average variance extracted (AVE)	≥0.5	Hair et al. (2019)
Heterotrait-monotrait (HTMT)	<0.9	Henseler et al. (2015)

Convergent validity was supported because all average variance extracted (AVE) values exceeded the recommended threshold of 0.50, ranging from 0.575 to 0.589. In addition, discriminant validity was established, as all heterotrait-monotrait (HTMT) ratios remained below the recommended threshold of 0.90 (Table 5). Overall, the results suggest that the measurement model demonstrated acceptable reliability and validity.

**Table 5:** Discriminant validity HTMT ratios.

Construct	FTPPCP	TS	WFC
Female teacher perceived career progression			
Technostress	0.581		
Work–family conflict	0.780	0.570	

### 4.4 Structural model

**Figure 5:**
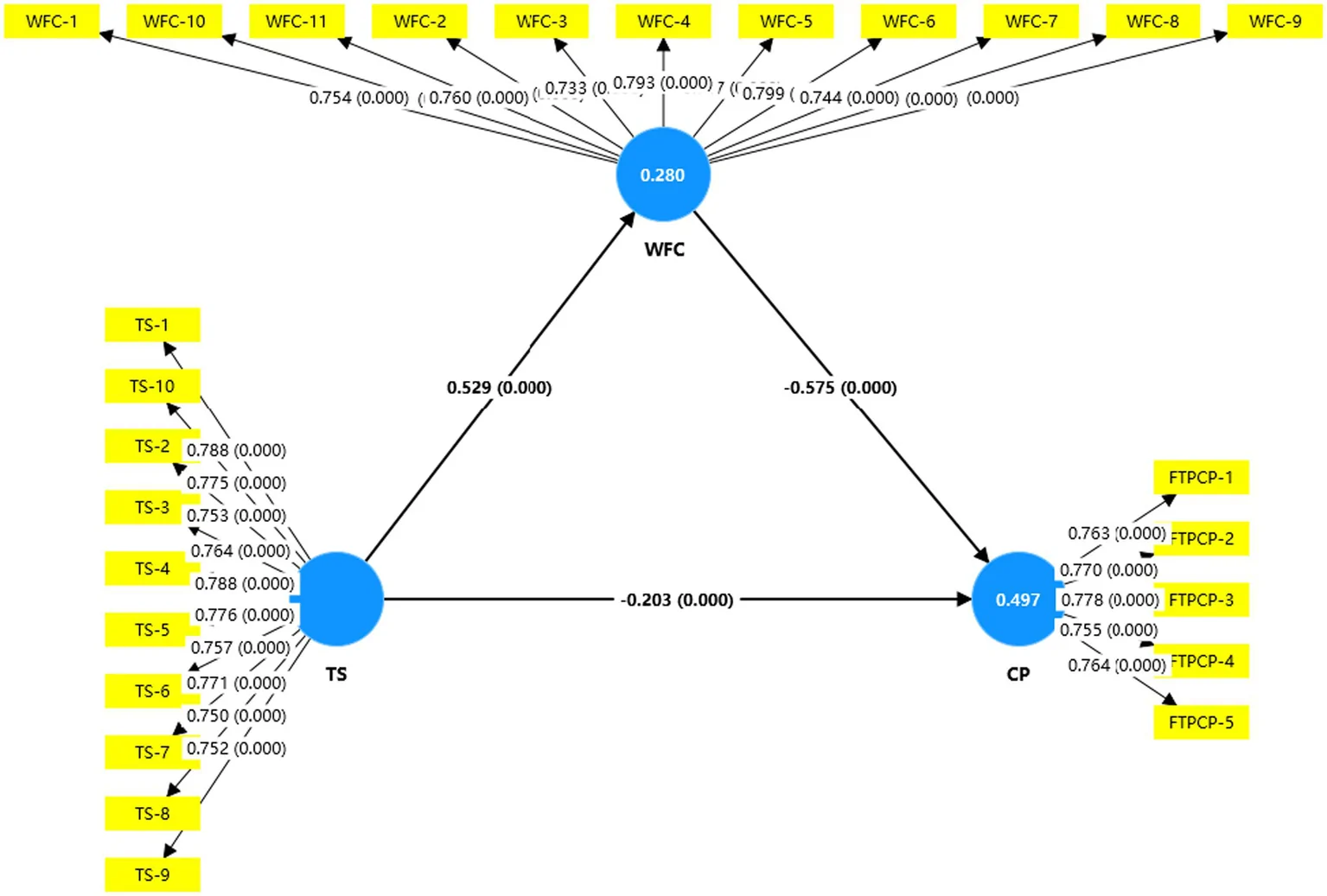
Path model.

In this study, perceived career progression refers to respondents’ subjective evaluation of their career-related achievement and progress at the time of the survey. Accordingly, the following results should not be interpreted as evidence of actual promotion, rank advancement, or longitudinal career development. After confirming the adequacy of the measurement model, the structural model was evaluated to test the hypothesized relationship between technostress, work–family conflict, and perceived career progression. The evaluation focuses on path coefficients, determination coefficients (*R*^2^), and predictive relevance (*Q*^2^). The *R*^2^ value is used to evaluate the explanatory power of endogenous structures, while the *Q*^2^ value obtained through the blindfold program is used to evaluate predictive relevance. The significance of the hypothesis relationship was examined using a bootstrap program with 5,000 bootstrap resamples. The results of the structural model evaluation are shown in Tables 6, 7, as well as Figure 5.

**Table 6:** Model explanatory and predictive relevance.

Construct	*R* ^2^	*Q* ^2^	Results
WFC	0.280	0.288	Modest/medium
FTPPCP	0.497	0.158	Moderate/medium

**Table 7:** Results of direct and indirect path analysis.

Path	Path coefficient	*t*-value	*p*-value	95% confidence	Results
				Interval [2.5, 97.5%]	
TS→PPCP	−0.203	5.346	0.000	[−0.277, −0.129]	H1: supported
WFC→PPCP	−0.575	16.746	0.000	[−0.64, −0.503]	H2: supported
TS→WFC→PCP	−0.305	11.912	0.000	[−0.357, −0.258]	H3: supported

As shown in Table 6, the *R*^2^ values for work–family conflict and perceived career progression were 0.280 and 0.497, respectively. These findings indicate modest explanatory power for work–family conflict and moderate explanatory power for perceived career progression (Hair et al., 2019). Regarding predictive relevance, the *Q*^2^ values for work–family conflict (0.288) and perceived career progression (0.158) were both above zero, suggesting that the model had acceptable predictive relevance. Based on the recommended guidelines, both endogenous constructs demonstrated medium predictive relevance (Hair et al., 2019).

In addition to *R*^2^ and *Q*^2^, effect sizes (*f*^2^) were calculated to evaluate the relative contribution of each predictor construct to the explained variance of the endogenous constructs, following Cohen’s (1988) guidelines (small ≥0.02, medium ≥0.15, large ≥0.35). As shown in Table 8, work–family conflict exerted a large effect on perceived career progression (*f*^2^ = 0.474), technostress exerted a large effect on work–family conflict (*f*^2^ = 0.389), while the direct effect of technostress on perceived career progression was small (*f*^2^ = 0.059). These results indicate that work–family conflict was the stronger direct predictor of perceived career progression.

**Table 8:** Effect sizes (*f*^2^) of predictor constructs.

Construct	*f* ^2^	Effect size
TS→PPCP	0.059	Large
TS→WFC	0.389	Small
WFC→PCP	0.474	Large

The bootstrapping results for the hypothesized associations are summarized in Table 7. The research model included three hypotheses concerning the associations among technostress, work–family conflict, and perceived career progression. All hypothesized paths were statistically significant, and the results for each path are reported below.

*H1*: Technostress is negatively associated with female teachers’ perceived career progression.

As shown in Table 7, the first hypothesis proposed a negative relationship between technostress and female teachers’ perceived career progression, and the results supported this hypothesis (*β* = −0.203, *t* = 5.346, *p* < 0.001). The 95% confidence interval [−0.277, −0.129] did not include zero, further confirming the significance of the relationship (Hair et al., 2019). These findings indicate that higher levels of technostress was significantly and negatively associated with female teachers’ perceived career progression.

This finding is consistent with research reporting associations between technostress and teachers’ professional engagement, job satisfaction, and participation in career-related activities (Marrinhas et al., 2023; Marchiori et al., 2020; Ibrahim et al., 2024; Camacho and Barrios, 2022). It suggests that teachers who report greater technology-related strain may also evaluate their current career progress less favorably. As the study used cross-sectional data, this finding should not be interpreted as evidence that technostress causes changes in actual career advancement.

*H2*: Work–family conflict is negatively associated with female teachers’ perceived career progression.

As shown in Table 7, work–family conflict was negatively associated with female teachers’ perceived career progression (*β* = −0.575), *t* = 16.746, *p* < 0.001). The 95% confidence interval [−0.640, −0.503] did not include zero. Thus, H2 was supported. Higher levels of work–family conflict were associated with less favorable subjective evaluations of career-related achievement and progress among female teachers.

This finding is consistent with previous research reporting negative associations between work–family conflict and career satisfaction or other subjective career-related outcomes (Adu et al., 2023). Studies in educational settings have also linked work–family conflict with role strain and less favorable work-related experiences among female teachers (Dlamini et al., 2024; Essiomle et al., 2024; Wang and Wareewanich, 2024). Given the cross-sectional design, the present result does not show that work–family conflict causes changes in actual career advancement.

*H3*: Work–family conflict statistically mediates the association between technostress and female teachers’ perceived career progression.

Table 9 presents the indirect association between technostress and perceived career progression through work–family conflict. The mediation analysis evaluated whether the association between technostress and female teachers’ perceived career progression was statistically accounted for, in part, by work–family conflict. The results of the indirect effect, total effect, and mediation analysis are reported below.

**Table 9:** Specific indirect effect with WFC as a mediator.

Path	Path coefficient	Mean	Standard deviation	*t*	*p*	95% confidence interval
TS→WFC→PPCP	−0.305	−0.306	0.026	11.912	0.000	[−0.357, −0.258]

#### 4.4.1 Indirect effects with WFC as a mediator

The indirect association between technostress and perceived career progression through work–family conflict is presented in Table 8. This indirect association was statistically significant (*β* = −0.305, *t* = 11.912, *p* < 0.001). The 95% confidence interval [−0.357, −0.258] did not include zero. Thus, the results were consistent with the proposed indirect association between technostress and perceived career progression through work–family conflict. Given the cross-sectional design, this finding should not be interpreted as evidence that technostress temporally increases work–family conflict or causes changes in career progression.

#### 4.4.2 Total association with work–family conflict as a mediator

Table 10 presents the total association between technostress and female teachers’ perceived career progression when work–family conflict was included in the model. Technostress was negatively associated with perceived career progression (*β* = −0.508, M = −0.510, SD = 0.035, *t* = 14.523, *p* < 0.001). The 95% confidence interval [−0.578, −0.439] did not include zero. The direct and indirect associations were both statistically significant, which was consistent with partial mediation by work–family conflict. These results concern respondents’ subjective evaluations of career-related achievement and progress, rather than actual promotion, academic-rank advancement, or longitudinal career development.

**Table 10:** Total effects of WFC as a mediator.

Path	Path coefficient	Mean	Standard deviation	*t*	*p*	95% confidence interval
TS→PPCP	−0.508	−0.510	0.035	14.523	0.000	[−0.578, −0.439]

#### 4.4.3 Indirect association through work–family conflict

Table 11 summarizes the indirect association through work–family conflict in the relationship between technostress and female teachers’ perceived career progression. The variance accounted for (VAF) was 60.04%, indicating that the indirect association through work–family conflict represented a substantial proportion of the total association between technostress and perceived career progression. Because both the direct and indirect associations were statistically significant, the results were consistent with partial mediation by work–family conflict. Given the cross-sectional design, the VAF should be interpreted as a descriptive indicator of the relative size of the indirect association rather than evidence of a temporal causal mechanism.

**Table 11:** Mediating effect of WFC.

Hypotheses	IV	MV	DV	Direct effect	Indirect effect	Total effect	VAF	Results
H3	TS	WFC	FTPPCP	−0.203	−0.305	−0.508	60.04%	

These findings are consistent with the proposition that technostress is negatively associated with female teachers’ perceived career progression through work–family conflict. They also accord with prior research linking technology-related work demands to work–family conflict and other work-related outcomes (Finstad et al., 2024; Shi et al., 2023). From a resource-based perspective, teachers experiencing greater technology-related strain may report that digital work demands require more time and psychological effort. Such experiences may coincide with greater work–family conflict and less favorable subjective evaluations of career-related achievement and progress. Previous studies have likewise reported associations between work–family conflict and career satisfaction, professional engagement, and other career-related perceptions (Gerekan et al., 2024; Qin et al., 2025; Keeler et al., 2024).

## 5 Discussion

This study examined the effects of technostress and work–family conflict on female teachers’ perceived career progression in Chinese higher education institutions. As digital technologies become increasingly integrated into teaching, research, and administrative work, female teachers are facing growing technological demands that may negatively affect their professional development and work-life balance. Drawing on the JD-R model and COR theory, this study found that technostress was negatively associated with female teachers’ perceived career progression, both directly and indirectly through work–family conflict. These findings suggest that excessive technological demands may create sustained pressure that limits female teachers’ ability to engage in perceived career progression activities and maintain work-family balance.

The first finding of this study is that technostress significantly reduced female teachers’ perceived career progression, which is consistent with previous studies reporting that technostress weakens teachers’ professional engagement and limits opportunities for perceived career progression (Riche et al., 2023; Pandit and Paul, 2023; Solís García et al., 2021). Female teachers may be particularly vulnerable to techno-overload and techno-complexity because they are often expected to adapt to multiple digital systems while simultaneously managing teaching, research, and family responsibilities (Boyer-Davis and Berry, 2022). From the perspective of COR theory, continuous technological demands may gradually consume teachers’ time, energy, and psychological resources, especially when institutional support and technical training are insufficient. As these resources become depleted, female teachers may experience lower motivation, reduced self-efficacy, and fewer opportunities to participate in career-enhancing activities such as research collaboration, academic networking, and leadership development. These findings indicate that higher education institutions should strengthen technical support systems, provide continuous digital skills training, and ensure a more balanced distribution of digital workloads to reduce the negative impact of technostress on female teachers’ perceived career progression.

The second finding was that work–family conflict was negatively associated with female teachers’ perceived career progression, which is consistent with previous studies reporting associations between work–family conflict and subjective career-related outcomes (Cao et al., 2025; Shreffler et al., 2019; Wang, 2023; Khan and Khan, 2022). In the Chinese higher education context, many female teachers continue to bear major family responsibilities, particularly childcare and eldercare, alongside their teaching and research duties. Although women’s participation in academia has increased considerably, traditional family expectations remain influential and often place greater domestic responsibilities on women. As a result, female teachers may have less time and energy available for research activities, conference participation, academic networking, and leadership development. Over time, persistent work–family conflict may reduce professional motivation and limit opportunities for perceived career progression. The findings also suggest that institutional support can help reduce the negative effects of work–family conflict. Flexible work arrangements, family-friendly policies, and supportive supervisors may help female teachers better manage competing responsibilities and maintain perceived career progression.

The third finding was that the indirect association between technostress and female teachers’ perceived career progression through work–family conflict was statistically significant. Continuous technological demands may require female teachers to spend additional time adapting to online teaching systems, administrative platforms, and digital communication tools. In many cases, technology-related work extends beyond regular working hours and gradually interferes with family life and personal time. As work-family boundaries become less clear, female teachers may experience higher levels of stress, fatigue, and role conflict. These pressures may eventually reduce motivation and limit participation in career-enhancing activities such as research collaboration, professional training, and leadership opportunities. This finding supports the view that technostress may hinder female teachers’ perceived career progression by intensifying work–family conflict and depleting personal resources over time.

This finding may be better understood within the context of Chinese higher education, where female teachers are facing increasing technological demands alongside heavy teaching responsibilities and family obligations. As universities continue to promote digital teaching and online administrative systems, many female teachers are required to spend additional time adapting to new technologies while still managing research and household responsibilities. Under these conditions, continuous exposure to technology-related demands may gradually increase work–family conflict and reduce the time and energy available for career development activities. From the perspective of the Job Demands-Resources (JD-R) model (Bakker and Demerouti, 2017), technostress can be viewed as a demanding work condition that becomes more difficult to manage when institutional support and career resources are insufficient. Previous studies have similarly shown that work–family conflict plays an important role in linking workplace stressors to career-related outcomes among female educators (Sarwar and Imran, 2019; Dong et al., 2025; Ghislieri et al., 2017; Sommovigo et al., 2023).

However, previous studies have reported different views regarding the relationship among technostress, work–family conflict, and perceived career progression. Some researchers have suggested that limited career advancement opportunities may increase work–family conflict, as female teachers who feel blocked in their professional development may devote more time and attention to family responsibilities (Zhang et al., 2022). Other studies have argued that family-related pressures may occur earlier than workplace stressors because family demands reduce the time and energy available to cope with work responsibilities. The differences between these findings and the present study may be related to differences in cultural and institutional contexts. In the Chinese higher education environment, the rapid expansion of digital teaching and administrative systems has increased the technological demands placed on university teachers. When these demands are not matched with sufficient institutional support, female teachers may experience greater difficulty balancing work and family responsibilities. Therefore, this study suggests that technostress may increase work–family conflict, which in turn negatively affects perceived career progression among female teachers.

Although technostress has been widely studied in relation to job stress, burnout, and wellbeing, fewer studies have examined its influence on perceived career progression among female teachers in higher education. Existing research has mainly examined the associations between technostress and short-term psychological outcomes. Comparatively less attention has been given to its association with perceived career progression among female teachers in higher education. In the Chinese academic context, these issues may become more prominent because rapid technological change is occurring alongside increasing academic expectations and continuing family responsibilities for women. As a result, understanding the relationship among technostress, work–family conflict, and perceived career progression is important for both future research and institutional practice. Higher education institutions may need to provide more effective technical support, clearer workload arrangements, and family-friendly policies to reduce the pressures experienced by female teachers. Addressing these issues may help support female teachers’ professional development and contribute to a more inclusive academic environment.

### 5.1 Theoretical implications

This study contributes to the literature on female teachers’ perceived career progression in several ways. First, it applies the Job Demands-Resources (JD-R) framework to explain how technostress may negatively influence perceived career progression among female faculty in higher education. The findings suggest that technology-related demands are not only associated with daily work pressure but may also affect longer-term professional development. Second, this study adds to the limited research examining technostress in relation to female teachers’ perceived career progression, particularly within the context of Chinese higher education. While previous studies have mainly focused on psychological wellbeing and job-related outcomes, this study further shows that technostress may also influence perceived career progression. Third, the findings highlight the mediating role of work–family conflict. The results indicate that technological demands may increase work–family conflict, which in turn reduces female teachers’ opportunities and motivation for perceived career progression. Overall, this study provides additional insight into how digital transformation may shape female faculty’s professional experiences and perceived career progression in higher education.

### 5.2 Practical implications

The findings suggest that higher education institutions may consider measures that reduce unnecessary technology-related demands and support employees’ management of work and family roles. Such measures may include accessible technical support, training for digital platforms, clearer expectations regarding online availability, and reasonable allocation of digital administrative tasks. Institutions may also consider flexible work arrangements and family-supportive practices. These measures are intended to support teachers’ work experiences and subjective career-related evaluations.

Higher education institutions can help reduce technostress by providing continuous technical training, accessible IT support, and clearer guidelines regarding the use of digital platforms. It is also important to avoid excessive technology-related workloads and unreasonable expectations for constant online availability outside regular working hours. At the same time, institutions should create more supportive environments for female teachers through flexible work arrangements, mentoring opportunities, family-friendly policies, and transparent promotion systems. These forms of support may help female faculty better manage work and family responsibilities while maintaining perceived career progression.

In addition, policymakers and university administrators should pay closer attention to gender differences in the experience of educational digitalization. As universities continue to expand the use of digital technologies, equal access to technological resources, professional training, and career opportunities becomes increasingly important. Reducing unnecessary administrative burdens and strengthening institutional support may help female teachers participate more fully in teaching, research, and leadership activities. In the long term, creating a more supportive academic environment may contribute not only to female teachers’ professional development but also to the sustainable development of higher education institutions.

These practical considerations are also relevant to Sustainable Development Goal 3 (Good Health and Well-being), Sustainable Development Goal 4 (Quality Education), and Sustainable Development Goal 5 (Gender Equality). By reducing unnecessary technology-related demands and supporting employees’ management of work and family roles, higher education institutions may help create healthier, more inclusive, and more sustainable academic workplaces.

### 5.3 Limitations and future research

This study has several limitations. First, the sample was limited to female teachers from higher education institutions in Henan Province, China, and was recruited through a non-probability sampling approach. The findings may therefore not be generalizable to male teachers, other regions of China, or other educational and cultural settings. Second, the study focused on technostress, work–family conflict, and perceived career progression. Other relevant factors, including institutional support, digital training opportunities, technological infrastructure, and individual coping resources, were not examined. Third, the cross-sectional design and use of a subjective career success scale do not permit conclusions about temporal ordering, causality, or actual career mobility. Perceived career progression reflected respondents’ subjective evaluations of career-related achievement and progress at the time of the survey, rather than objective indicators such as promotion, academic-rank advancement, salary growth, or changes in professional responsibilities. Fourth, most items in the work–family conflict measure referred broadly to family activities, family responsibilities, or family life. However, one item explicitly referred to becoming a better parent or spouse and may have been less applicable to respondents who were unmarried or did not have children. Marital status was reported descriptively but was not included as a control variable or examined through a sensitivity analysis. The findings concerning work–family conflict should therefore be interpreted with caution.

Future research could use longitudinal designs and combine subjective career evaluations with objective indicators, such as promotion records, academic rank, salary progression, publication output, or leadership appointments. Studies could also examine whether the findings differ across marital, parenting, institutional, and regional groups. Qualitative and mixed-methods research may further clarify how teachers understand technology-related demands and family responsibilities in their daily work. Finally, future studies could include institutional support, digital self-efficacy, flexible work arrangements, and coping strategies to provide a broader account of the associations among technostress, work–family conflict, and perceived career progression.

## 6 Conclusion

This study examined the associations among technostress, work–family conflict, and perceived career progression among female teachers in higher education institutions in Henan Province, China. The findings showed that technostress was negatively associated with perceived career progression, both directly and indirectly through work–family conflict. In this study, perceived career progression referred to respondents’ subjective evaluations of their career-related achievement and progress at the time of the survey; it did not measure actual promotion, academic-rank advancement, or long-term career mobility. By focusing on female teachers in an inland Chinese higher education context, this study applies the Job Demands-Resources model and Conservation of Resources theory to interpret the observed associations among technology-related demands, work–family conflict, and subjective career-related evaluations. The findings suggest that universities may consider strengthening technical support, clarifying expectations regarding digital work, and providing family-supportive arrangements. Such measures may help improve teachers’ experiences of technology-related work demands and work–family conflict.

## Data Availability

The raw data supporting the conclusions of this article will be made available by the authors, without undue reservation.
