# Supplementary material for: The impact of technostress on female teachers’ perceived career progression: the mediating role of work–family conflict
Source: Front Psychol. 2026 Aug 20;17:1881723. doi: 10.3389/fpsyg.2026.1881723 (PMC13537942; doi:10.3389/fpsyg.2026.1881723)
Supplement: Supplementary file 1 [file Supplementary_file_1.DOCX]

**Appendix 1**

**Technostress：** Technostress Creator Scale (Ragu-Nathan et al., 2008)

Instructions: Please answer the following questions about yourself by indicating the extent of your agreement using the following Likert scale.

| **Strongly Disagree** | **Disagree** | **Neither agree not disagree** | **Agree** | **Strongly Agree** |
| --- | --- | --- | --- | --- |
| **1** | **2** | **3** | **4** | **5** |

| **No.** | **Items** | **Likert Scale** | | | | |
| --- | --- | --- | --- | --- | --- | --- |
| 1 | I am forced by this technology to do more work than I can handle. | 1 | 2 | 3 | 4 | 5 |
| 2 | I am forced to change my work habits to adapt to new technologies. | 1 | 2 | 3 | 4 | 5 |
| 3 | I have a higher workload because of increased technology complexity. | 1 | 2 | 3 | 4 | 5 |
| 4 | I spend less time with my family due to this technology. | 1 | 2 | 3 | 4 | 5 |
| 5 | I have to sacrifice my vacation and weekend time to keep current on new technologies. | 1 | 2 | 3 | 4 | 5 |
| 6 | I feel my personal life is being invaded by this technology. | 1 | 2 | 3 | 4 | 5 |
| 7 | I do not know enough about technology to handle my job satisfactorily. | 1 | 2 | 3 | 4 | 5 |
| 8 | I need a long time to learn how to use new technologies. | 1 | 2 | 3 | 4 | 5 |
| 9 | I do not find enough time to study and update my technology skills. | 1 | 2 | 3 | 4 | 5 |
| 10 | There are constant technological updates that I must learn to manage. | 1 | 2 | 3 | 4 | 5 |

**Appendix 2**

**WORK-FAMILY CONFLICT:**Work-family conflict scale （Carlson et al. 2000）

| **Strongly Disagree** | **Disagree** | **Neither agree not disagree** | **Agree** | **Strongly Agree** |
| --- | --- | --- | --- | --- |
| **1** | **2** | **3** | **4** | **5** |

| **No.** | **Items** | **Likert Scale** | | | | |
| --- | --- | --- | --- | --- | --- | --- |
| 1 | The time I must devote to my job keeps me from participating equally in household responsibilities and activities. | 1 | 2 | 3 | 4 | 5 |
| 2 | I have to miss family activities due to the amount of time I must spend on work responsibilities. | 1 | 2 | 3 | 4 | 5 |
| 3 | The time I spend on family responsibilities often interfere with my work responsibilities. | 1 | 2 | 3 | 4 | 5 |
| 4 | The time I spend with my family often causes me not to spend time in activities at work that could be helpful to my career. | 1 | 2 | 3 | 4 | 5 |
| 5 | When I get home from work I am often too frazzled to participate in family activities/ responsibilities. | 1 | 2 | 3 | 4 | 5 |
| 6 | I am often so emotionally drained when I get home from work that it prevents me from contributing to my family. | 1 | 2 | 3 | 4 | 5 |
| 7 | Due to all the pressures at work, sometimes when I come home I am too stressed to do the things I enjoy. | 1 | 2 | 3 | 4 | 5 |
| 8 | Because I am often stressed from family responsibilities, I have a hard time concentrating on my work. | 1 | 2 | 3 | 4 | 5 |
| 9 | Tension and anxiety from my family life often weakens my ability to do my job. | 1 | 2 | 3 | 4 | 5 |
| 10 | The problem-solving behaviors I use in my job are not effective in resolving problems at home. | 1 | 2 | 3 | 4 | 5 |
| 11 | The behaviors I perform that make me effective at work do not help me to be a better parent and spouse. | 1 | 2 | 3 | 4 | 5 |

**SECTION G: CAREER PROGRESSION**

G. **CAREER PROGRESSION:**Career Success Scale (CPS) Judge et al. (1994) Cronbach’s Alpha =0.926

| Strongly Disagree | Disagree | Neither agree not disagree | Agree | Strongly Agree |
| --- | --- | --- | --- | --- |
| 1 | 2 | 3 | 4 | 5 |

| **No.G** | **Items** | Likert Scale | | | | |
| --- | --- | --- | --- | --- | --- | --- |
| 1 | I am satisfied with the success I have achieved in my career. | 1 | 2 | 3 | 4 | 5 |
| 2 | I am satisfied with the progress I have made toward meeting my overall career goals. | 1 | 2 | 3 | 4 | 5 |
| 3 | I am satisfied with the progress I have made toward meeting my goals for advancement. | 1 | 2 | 3 | 4 | 5 |
| 4 | I am satisfied with career progress I have made toward meeting my goals for income. | 1 | 2 | 3 | 4 | 5 |
| 5 | I am satisfied with the career progression I have made toward meeting my goals for the development of new skills. | 1 | 2 | 3 | 4 | 5 |
